# Relationship between berry intake and frailty in the elderly and the mediating role of six blood markers: based on Chinese and American studies

**DOI:** 10.3389/fnut.2026.1823930

**Published:** 2026-07-10

**Authors:** Ruolin Zhang, Yongyao Du, Jingping Li, Lili Ma, Jinlin Yan, Xiaoyan Zeng, Manping Zhou, Chao Kang, Xiaoli Peng

**Affiliations:** 1 Department of Clinical Nutrition, The Third Affiliated Hospital of Chengdu Medical College Chengdu Pidu District People’s Hospital, Chengdu, Sichuan, China; 2 School of Public Health, Chengdu Medical College, Chengdu, Sichuan, China; 3 Department of Clinical Nutrition, The General Hospital of Western Theater Command, Chengdu, Sichuan, China; 4 Sichuan Provincial Key Laboratory of Philosophy and Social Sciences for Intelligent Medical Care and Elderly Health Management, Chengdu Medical College, Chengdu, Sichuan, China

**Keywords:** berry intake, blood markers, China, elderly people, frailty, NHANES

## Abstract

**Background:**

Frailty has become a major public health challenge with the acceleration of global aging. Berries are rich in bioactive components, but the mechanism and population-specific association with frailty is not clear.

**Methods:**

For Chinese populations, data from hospitalized patients at Pidu District People’s Hospital during May–July 2024 were analyzed using the Frailty phenotype (FP) to evaluate frailty. This study employed the 24-h dietary recall method from the 2003–2008 National Health and Nutrition Examination Survey (NHANES) database to assess berry consumption. A frailty index (FI) was calculated using approximately 49 indicators to determine frailty status. The research utilized a weighted regression model to investigate the relationship between berry intake and frailty severity, while examining the mediating role of six blood biomarkers through mediation analysis.

**Results:**

This study enrolled 357 Chinese elderly individuals and 5,553 American adults aged 65 and older. The findings in China demonstrated that berry consumption was associated with reduced frailty risk. This association remained statistically significant even after adjusting for other potential confounding factors. In the NHANES, compared to individuals who did not consume berries, daily berry intake ranging from 37–74 grams and 74–158 grams was associated with reduced risks of frailty, and these associations remained statistically significant after adjustment for other confounding factors. Notably, this association was more pronounced among males. Mediation analysis indicated that HbA1c levels mediated 6.64% of the effect.

**Conclusion:**

This study demonstrates that berry consumption is associated with a lower risk of frailty. Particularly for males, incorporating berries into dietary intervention strategies targeting this population may offer potential benefits; additionally, glycated hemoglobin (HbA1c) could serve as an indicator for efficacy monitoring in subsequent interventional studies.

## 1 Introduction

Frailty is a core syndrome in the aging process, characterized by a decline in physiological reserves, dysfunction of multiple systems, and reduced stress resistance (1). This significantly increases the risk of disability, hospitalization, and death in the elderly (2). Epidemiological data show that the prevalence of weakness among people aged 50 and older ranges from 26.5 to 38.9% (3). The prevalence sharply increases with age, reaching 41.9% in those over 70 (4). As the global population structure ages at an accelerating rate, preventing and delaying frailty has become a major public health challenge.

Diet quality, an important lifestyle factor that can be influenced, is closely linked to the development of frailty (5). Previous studies have demonstrated that elderly males with a high-fat, low-fiber dietary pattern are more prone to frailty (6). Moreover, the dietary quality of the elderly in the pre-frailty stage exhibits poorer balance (7). These studies collectively suggest a close relationship between diet and frailty, indicating that dietary interventions may be considered to improve frailty.

Among various nutritional intervention strategies, berry fruits (such as blueberries, strawberries, and raspberries) have gained significant attention due to their high content of anthocyanins, flavonoids, and dietary fiber (8). In the middle-aged and elderly population, studies have found a U-shaped association between the intake of total flavonoids and various subclasses of flavonoids and physical frailty (9). However, no association was found between flavonoid intake and frailty in the adult cohort of the Framingham Cohort (10). Therefore, high-quality, large-sample epidemiological studies directly linking berry intake to frailty remain relatively scarce, necessitating further exploration.

It is important to note that the pathological essence of frailty is a complex interaction resulting from dysfunctions in multiple physiological systems, involving imbalances in key physiological processes such as metabolic homeostasis (e.g., blood glucose and lipid regulation), nutritional status, renal function, and oxidative stress/inflammatory responses (11). These systemic dysfunctions can often be reflected through specific blood biomarkers. For example, hemoglobin A1c (HbA1c) is a core indicator reflecting long-term blood glucose control and insulin resistance (12). Liprisk [such as total cholesterol (TC) and triglycerides (TG)] are closely linked to cardiovascular metabolic risk (13, 14). Albumin (ALB) is an important marker for assessing nutritional reserves and protein synthesis status (15). Creatinine (CRE) levels primarily reflect the baseline renal function (16). And uric acid (UA) is associated with purine metabolism, oxidative stress, and inflammatory states (17). These biomarkers have been shown to be associated with frailty risk to varying degrees.

To investigate the association between berry consumption and frailty status in elderly individuals and its underlying biological mechanisms, this study utilized data from patient records from Pidu District People’s Hospital in China and the NHANES. Through cross-sectional analysis, we evaluated the correlation between dietary berry intake and frailty status among adults aged 65 and older, while systematically examining the mediating roles of six blood biomarkers (TC, UA, ALB, CRE, HbA1c, TG) in the relationship between berry consumption and frailty. This study aims to validate the following three primary hypotheses separately in two distinct populations: (1) Among individuals aged 65 and older, berry consumption may be negatively associated with the risk of frailty, and this association persists after controlling for multiple confounding factors; (2) The negative correlation between berry consumption and frailty may vary across different subgroups; and (3) Six blood biomarkers may play a mediating role between berry consumption and frailty.

## 2 Methods

### 2.1 Study population

#### 2.1.1 Chinese study in Pidu

**Figure 1:**
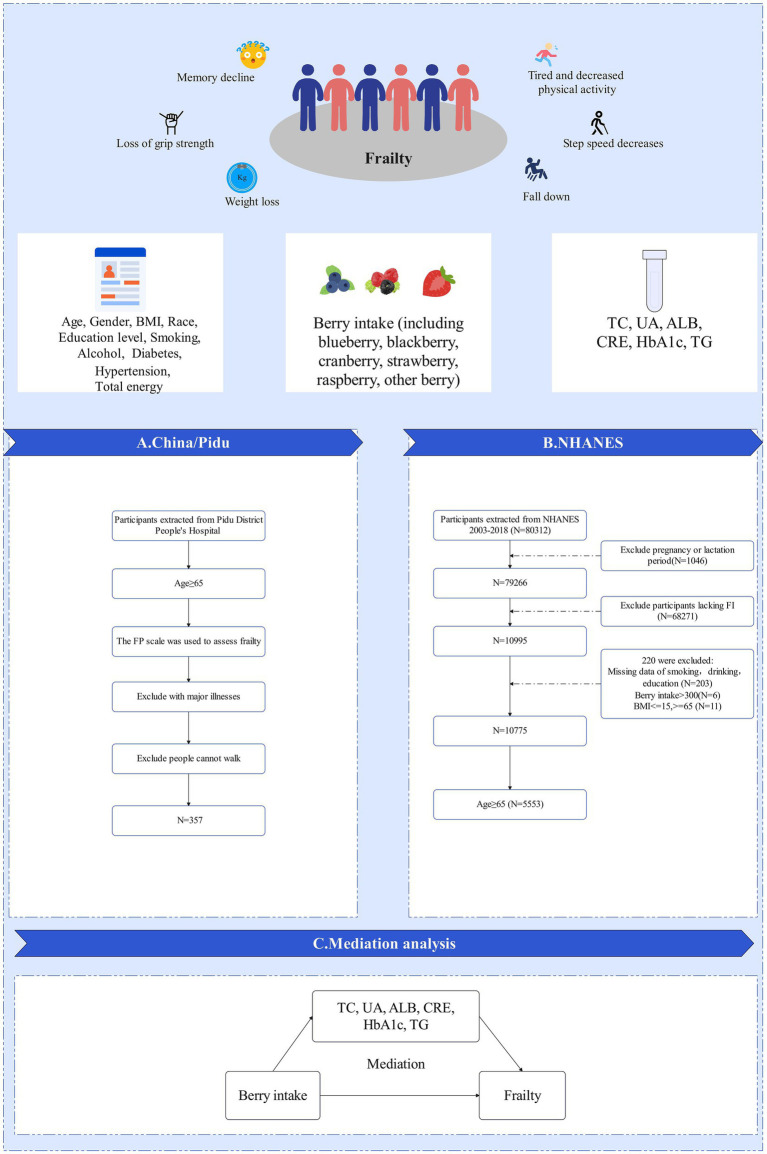
Flow chart of study design.

This study is divided into two phases, as illustrated in Figure 1. In the first stage, people were selected by completely random sampling method from Pidu District People’s Hospital, Inclusion criteria were: (1) Age 65 or older, (2) complete berry intake records, and (3) Complete frailty scale assessment results. Excluding those with major illnesses and those who with impossibility of walking. Major diseases are defined as conditions such as malignant arrhythmias, heart failure of grade III or higher, cerebral hemorrhage, or aortic dissection, which render patients unsuitable for participation in the study. “Impossibility of walking” is clearly defined as: the inability to walk independently for 10 meters due to any cause (e.g., severe osteoarthritis, nonunion of fractures, paraplegia, advanced neuromuscular disorders, etc.), regardless of the use of assistive devices such as crutches or walkers, or the requirement for continuous assistance from others for mobility. This study has been approved by the Medical Ethics Committee of Pidu Municipal People’s Hospital (Approval number No.35,2024) and China Clinical Trial Registration Center number (ChiCTR2500096215). The calculation was performed using the “Sample Size Estimation for Estimating Population Rate” program in PASS2021 software. The required sample size was calculated to be 281 cases.

#### 2.1.2 NHANES study

In the second phase, we conducted an observational study using NHANES data, designed by the U. S. Centers for Disease Control and Prevention (CDC). And it is conducted every 2 years, aims to assess the nation’s health and nutrition status. The NHANES database includes survey data, laboratory results, physical examination findings, food and nutritional supplement information, and demographic statistics. In this study, we selected eight NHANES cycles from 2003 to 2018. Inclusion criteria were: (1) age over 65, (2) complete berry intake records, and (3) relatively complete frailty records (participants who answered at least 80% of the items). Exclusions included those who were breastfeeding or pregnant, individuals with an extreme BMI below 15 or above 65. Ultimately, 5,553 participants were included in this survey. In addition, given the increased prevalence of frailty with age, we divided the age group into two subgroups: 65–75 years and 75 years and above, for further exploratory analysis.

### 2.2 Assessment of frailty index

The clinical application in hospitals utilizes the Fried phenotype of frailty (FP) (18). The scale comprises five key criteria: (1) Weight loss exceeding 4.54 kg over the past year; (2) Weak grip strength (male ≤26 kg, female ≤18 kg); (3) Self-reported fatigue requiring significant effort for daily activities; (4) Slowed gait (walking 5 m takes >6 s); (5) Reduced physical activity (calorie expenditure) below <383 kcal/week for males and <270 kcal/week for females. Elderly individuals meeting 1–2 criteria are considered pre-frail, while those with morcriteriacriteria are classified as frail. This study defines frailty as the presence of at least 3 criterion. In NHANES, to ensure the accuracy of the frailty diagnosis in this study, we adopted strict data inclusion criteria. Our frailty index, consistent with previous research findings, requires a large number of variables (approximately 49) for reliable analysis (19). Frailty is quantified using the cumulative defect method. We calculated the frailty score by summing specific defect items and then dividing the total by the number of considered items, resulting in a score from 0 to 1, which reflects the degree of frailty, where 0 indicates no defects and 1 indicates complete frailty. A frailty index greater than 0.21 is considered indicative of frailty. The weakness scale is shown in Supplementary Table S1.

### 2.3 Berry intake

In our Chinese hospital population, berry intake was mainly based on a questionnaire “Have you eaten blueberries or other berry foods in the past month?.” In the data from NHANES, all participants were allowed to participate in two 24-h dietary recall interviews. The first interview took place at the NHANES Mobile Examination Center (MEC). Three to ten days later, the second interview was conducted by telephone. Each food or drink consumed by the participant had a unique code, and the interview also recorded the occasion, time, weight, and other relevant information. Dietary information was meticulously documented. The composition of each food and drink was calculated based on the USDA Food and Nutrition Database. By querying the food codes provided by NHANES, we determined whether the participants consumed berries. In this study, berry consumption was averaged over the two recall periods (if only one day’s data was available, that day’s data was used instead of the average). First, we divided the participants into two groups based on whether they ate berry or not. Subsequently, participants were categorized based on the interquartile range of berry intake. Finally, all participants (including those who did not consume berries) were divided into five groups: (1) 0 g/day; (2) >0 to ≤37 g/day; (3) >37 to ≤74 g/day; (4) >74 to ≤158 g/day; (5) >158 g/day. Day 1 dietary weights were used to adjust for unequal probabilities of selection among the respondents who only reported first dietary recalls, whereas day 2 dietary weights were used to adjust for additional nonresponse of the second recall among the respondents who provided 2 dietary recalls, as suggested by the NHANES analytical guidelines.

### 2.4 Covariates

We also investigated several potential confounding factors. We collected demographic data through standardized questionnaires and face-to-face interviews, including age, race, education level, total energy, smoking status, and alcohol consumption. BMI was determined based on a Physical Examination according to standard classification, which is divided into three categories: (1) normal weight (18.5–24.9), (2) underweight (18.5), and (3) overweight (≥25.0). Additionally, we examined the participants “history of two chronic diseases. Participants were diagnosed with diabetes if they answered” yes “to the question’ Was your doctor informed that you have diabetes?” in the Diabetes Questionnaire (DIQ); had fasting blood glucose levels of ≥7.0 mmol/L; had 2-h blood glucose levels of ≥11.1 mmol/L in the Oral Glucose Tolerance Test (OGTT); or had a glycated hemoglobin level of ≥6.5. The diagnosis of hypertension followed a similar method but was based on answers from the Blood Pressure and Cholesterol Questionnaire (BPQ).

### 2.5 Statistical analysis

Due to the specific sampling methods used in the survey, NHANES created multiple sample weights for analysis. Therefore, we integrated the sample weights of dietary intake data from different research periods into our analysis method. According to the NHANES analysis guidelines, when merging data from eight 2-year cycles, the original two-year sample weights were divided by 8 to create new sample weights (20). All tests were conducted as two-sided tests, with a significance level set at *p* < 0.05. All data analyses were performed using R studio (version 4.4.0).

When analyzing the baseline data of frail and non-frail populations, continuous variables were tested using *t*-tests or non-parametric tests, while categorical variables were tested using chi-square tests. We estimated the odds ratios (ORs) and their 95% confidence intervals (CIs) for the association between berry intake and frailty using weighted multivariate logistic regression models. These models included two adjusted models (Models II and III) and one unadjusted model (Model I). In Model II, we adjusted for age, BMI, and Gender. In Model III, we further adjusted for education level, smoking, alcohol consumption, total energy intake, diabetes and hypertension, while in the Chinese population, we further adjusted for education level, smoking, alcohol consumption, diabetes and hypertension in Model III. We analyzed berry intake as both a continuous and a categorical variable. We conducted subgroup analyses to explore whether the association between berry intake and frailty has significant interactions with factors such as age, gender, education level, BMI groups, etc. All subgroup analyses are exploratory in nature, aimed at generating hypotheses rather than providing confirmatory conclusions.

### 2.6 Mediating variables

Six blood biomarkers that may be associated with frailty were selected in this study: TC, UA, ALB, CRE, HbA1c, and TG. The collection and processing of serum samples were conducted by the mobile health examination center. To explore the relationship between blood biomarkers and frailty, we used univariate and multivariate logistic regression analysis. Additionally, we conducted univariate and multivariate linear regression analysis to investigate the relationship between berry intake and blood biomarkers. Causal mediation analysis was conducted using the mediation package. Mediating variables were analyzed with linear regression models, while the outcome variable (Frailty) was examined using a probit regression model, both adjusted for confounding factors such as age, gender, BMI, and total energy intake. The main findings include the direct relationship between berry intake and frailty, the mediating role of blood biomarkers, and the chain-mediated effect between berry intake and frailty. Indirect effects (ACME) were estimated via the product coefficient method combined with non-parametric Bootstrap (5,000 repetitions), with proportional mediation effects (PM) and their 95% confidence intervals reported.

## 3 Results

### 3.1 Baseline characteristics

**Figure 2:**
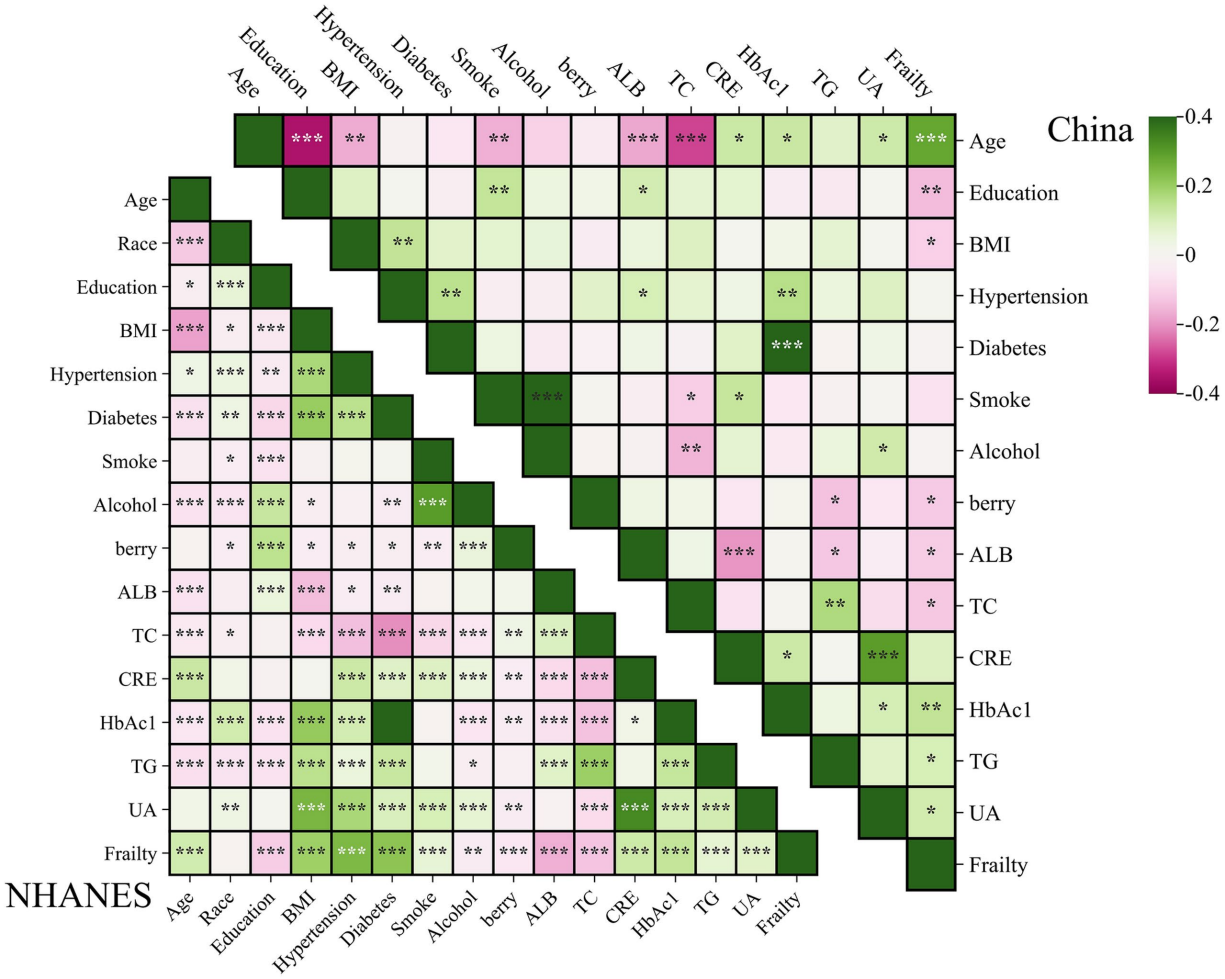
The correlation heatmap.

According to the study, China’s population shows a 19.33% prevalence rate (69/357), with about 45% of participants regularly consuming berries. Patients with frailty in China were generally older, had a lower BMI, and lower education levels. Supplementary Table S2 provides detailed baseline information for each participant. While the incidence of frailty in the U. S. population was 38.53% (2,140/5,553). Approximately 9.09% of participants had a habit of consuming berries. Overall, frailty risk was significantly associated with age (*p* < 0.001), gender (*p* < 0.001), education level (*p* < 0.001), smoking (*p* < 0.001), alcohol consumption (*p* < 0.001), total energy (*p* = 0.008), hypertension (*p* < 0.001), and diabetes (*p* < 0.001). Frail patients were typically older, had higher BMI, lower education levels, lower energy intake and higher rates of hypertension and diabetes. The correlation heatmap is shown in Figure 2.

### 3.2 The association between berry intake and frailty

This study investigates the association between berry consumption and frailty status through three models. First, we analyzed berry intake in Chinese populations. Compared to those who did not eat berries, with berries consistently showing a reduced frailty risk in all three models (*p* < 0.05). In NHANES, compared to individuals who do not consume berries, all three models demonstrated a significantly reduced risk of frailty among berry consumers (*p* < 0.05). Linear regression analysis was conducted on different categories of berry intake. The results showed that compared to those who did not consume berries, individuals consuming 37–74 grams of berries daily had a significantly reduced risk of frailty in the crude model (OR = 0.59, 95% CI: 0.41–0.86). Even after adjustment in model II (OR = 0.58, 95% CI: 0.40–0.85) and model III (OR = 0.61, 95% CI: 0.41–0.90), this association remained significant. In all three models, consuming over 74 grams or less than 158 grams of berries daily was associated with a statistically significant reduction in the risk of frailty. No statistically significant differences were observed among individuals who consumed more than 158 grams of berries. Finally, our results show a significant correlation between berry intake and frailty status, which is true for all berry intake categories (model I trend *p* value = 0.041, model II trend *p* value = 0.019, model III trend *p* value = 0.035). See Table 1 for details.

**Table 1:** Association between berry intake and frailty in people older than 65 years, weighted.

	Model 1		Model 2		Model 3	
	OR (95%CI)	*P*	OR (95%CI)	*P*	OR (95%CI)	*P*
Variables (China)						
Non-Berry intake	Ref		Ref		Ref	
Berry intake	0.53 (0.30 ~ 0.91)	0.024	0.51 (0.27 ~ 0.90)	0.022	0.51 (0.28 ~ 0.90)	0.023
Variables (NHANES)						
Non-Berry intake	Ref		Ref		Ref	
Berry intake	0.78 (0.66 ~ 0.93)	0.005	0.76 (0.64 ~ 0.90)	0.002	0.81 (0.68 ~ 0.96)	0.015
Berry groups						
=0	Ref		Ref		Ref	
>0, ≤37	0.90 (0.64 ~ 1.27)	0.600	0.87 (0.61 ~ 1.24)	0.400	0.92 (0.66 ~ 1.30)	0.600
>37, ≤74	0.59 (0.41 ~ 0.86)	0.007	0.58 (0.40 ~ 0.85)	0.005	0.61 (0.41 ~ 0.90)	0.014
>74, ≤158	0.69 (0.51 ~ 0.94)	0.018	0.71 (0.54 ~ 0.93)	0.012	0.72 (0.53 ~ 0.98)	0.036
>158	1.06 (0.76 ~ 1.48)	0.700	1.02 (0.73 ~ 1.43)	0.900	1.11 (0.82 ~ 1.58)	0.500
*P* for trend		0.041		0.019		0.035

Model II adjusted for BMI, age, gender, Model III adjusted for BMI, age, gender, education (China); Model III adjusted for BMI, age, gender, education, smoking, alcohol, total energy, hypertension and diabetes (NHANES).

### 3.3 Subgroup analysis for the association between berry intake and frailty

Furthermore, we conducted detailed subgroup analyses in NHANES, total energy intake was also included as a covariate. As shown in Supplementary Table S3, the association between berry intake and frailty was not significantly different among different BMI, gender, education level, smoking, alcohol consumption, diabetes, and hypertension. Our study only found that the association between berry intake and frailty was observed in the male (OR = 0.77, 95%CI:0.60–0.99). However, no such association was found in the subgroup analysis of the Chinese population. See Supplementary Table S4 for details.

Subsequently, we categorized the age groups into two categories: 65–75 years and 75 years and above, and conducted an exploratory analysis. The subgroup analysis revealed a link between berry intake and frailty in men. We then added other subgroups, including those with a history of alcohol consumption, and those with a history of diabetes, to the study. The results showed that in overweight men aged 75–85, berry intake was associated with a reduced risk of frailty (OR = 0.43, 95% CI: 0.21–0.89). Notably, similar associations were observed among overweight men who drank alcohol (OR = 0.37, 95% CI: 0.18–0.78). No correlation was found in overweight diabetic patients. And none of these associations have been found in women. For more details, see Table 2 and Supplementary Table S5. Similarly, no interesting associations were observed in the Chinese population, as detailed in Supplementary Table S6.

**Table 2:** Association between berry intake and frailty, stratified by age, and then was analyzed with gender, BMI, race, alcohol and smoking, weighted.

Variables	65–75		75–85	
	OR (95%CI)	*P*	OR (95%CI)	*P*
Male + overweight	0.61 (0.31 ~ 1.21)	0.200	0.43 (0.21 ~ 0.89)	**0.023**
Male + overweight + alcohol				
Yes	0.54 (0.26 ~ 1.12)	0.100	0.37 (0.18 ~ 0.78)	**0.009**
Male + overweight + diabetes				
Yes	0.59 (0.20 ~ 1.81)	0.400	0.72 (0.19 ~ 2.69)	0.600

All of the above were adjusted for covariates. Bold values indicate statistically significant associations at *P* < 0.05.

### 3.4 Mediation analysis

**Figure 3:**
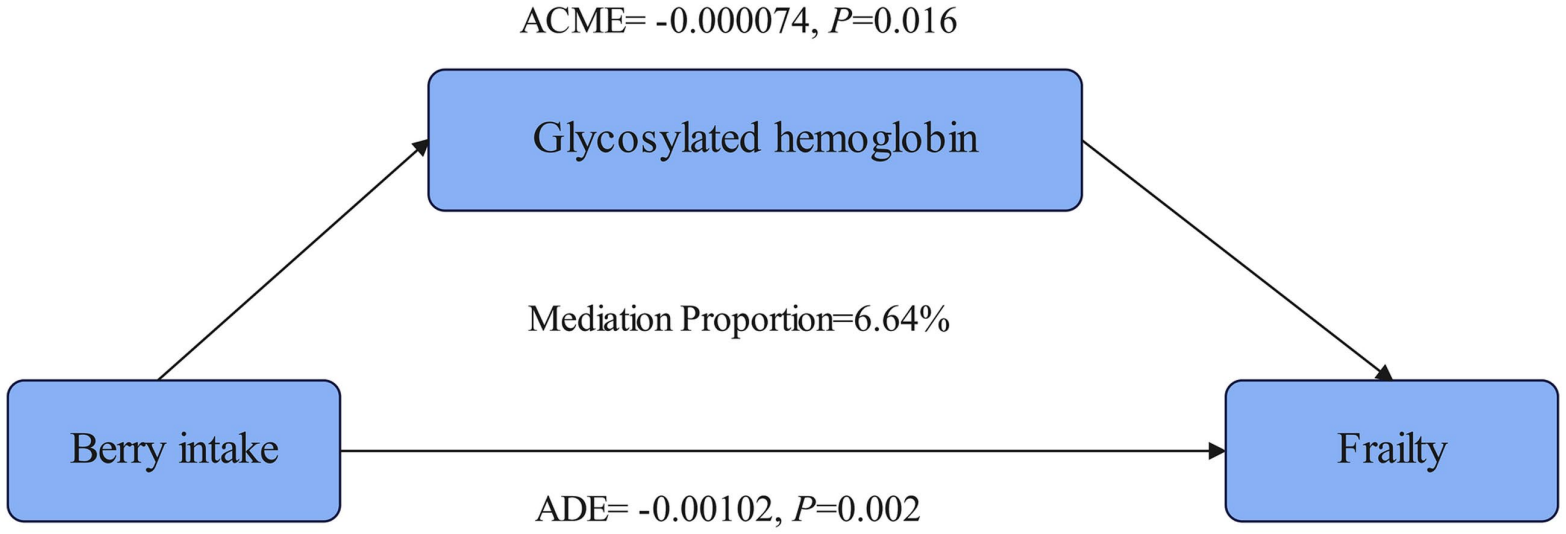
Mediation analysis of HbA1c as mediators between berry and frailty in the NHANES.

Table 3 presents the results of the NHANES multiple linear regression analysis. Berry intake showed a significant negative correlation with HbA1c, CRE, and UA. However, after adjusting for all variables in Model III, the significant negative correlation with UA was no longer significant. Similarly, Table 4 shows the results of the NHANES multivariate regression analysis. HbA1c and UA exhibited significant correlations with frailty. But when all variables were adjusted in Model III, this significant positive correlation no longer emerged. Notably, four indicators—ALB, TC, CRE, and TG—maintained significant associations with frailty status across all three models, demonstrating strong correlations. Supplementary Tables S7, S8 present the results of multivariate regression and multiple linear regression analyses for the Chinese population. Among these, only berry intake showed a significant negative correlation with TG across all three models, while HbA1c demonstrated significant correlations with frailty status in all three models. Among the six blood markers, the study found that HbA1c mediated the effect of berry on frailty in the NHANES was 6.64% (Figure 3; Supplementary Table S9). However, this effect has not been found in the Chinese population. Detailed data are shown in the Supplementary Table S10.

**Table 3:** Multiple linear regression analysis of berry intake and six blood markers in the NHANES.

NHANES	Model 1		Model 2		Model 3	
	Exp(β) (95%CI)	*P*	Exp(β) (95%CI)	*P*	Exp(β) (95%CI)	*P*
HbA1c	0.9992 (0.9984 ~ 0.9999)	0.029	0.9992 (0.9985 ~ 0.9999)	0.030	0.9993 (0.9986 ~ 0.9999)	0.021
ALB	1.0030 (0.9995 ~ 1.0070)	0.091	1.0030 (0.9993 ~ 1.0067)	0.110	1.0027 (0.9992 ~ 1.0065)	0.200
TC	1.0020 (0.9996 ~ 1.0040)	0.110	1.0014 (0.9996 ~ 1.0032)	0.140	1.0011 (0.9994 ~ 1.0029)	0.200
CRE	0.9474 (0.9161 ~ 0.9797)	0.002	0.9561 (0.9257 ~ 0.9875)	0.007	0.9644 (0.9333 ~ 0.9964)	0.030
TG	0.9989 (0.9977 ~ 1.0000)	0.083	0.9989 (0.9977 ~ 1.0001)	0.064	0.9991 (0.9979 ~ 1.0003)	0.130
UA	0.8702 (0.7699 ~ 0.9835)	0.026	0.8834 (0.7881 ~ 0.9902)	0.034	0.9077 (0.8066 ~ 1.0215)	0.110

**Table 4:** Multiple regression analysis of six blood markers and frailty in the NHANES.

Variables	Model 1		Model 2		Model 3	
	OR (95%CI)	*P*	OR (95%CI)	*P*	OR(95%CI)	*P*
HbA1c	1.8860 (1.6350 ~ 2.1760)	<0.001	1.8128 (1.5591 ~ 2.1077)	<0.001	1.2054 (0.9857 ~ 1.4742)	0.069
ALB	0.8883 (0.8627 ~ 0.9146)	<0.001	0.9005 (0.8736 ~ 0.9283)	<0.001	0.8993 (0.8720 ~ 0.9274)	<0.001
TC	0.8179 (0.7580 ~ 0.8825)	<0.001	0.7681 (0.7035 ~ 0.8387)	<0.001	0.8605 (0.7871 ~ 9,407)	0.001
CRE	1.0080 (1.0050 ~ 1.0110)	<0.001	1.0105 (1.0072 ~ 1.0138)	<0.001	1.0076 (1.0046 ~ 1.0106)	<0.001
TG	1.2120 (1.1280 ~ 1.3020)	<0.001	1.1855 (1.0959 ~ 1.2825)	<0.001	1.0927 (1.0032 ~ 1.1902)	0.042
UA	1.0001 (1.0001 ~ 1.0002)	0.002	1.0015 (1.0005 ~ 1.0024)	0.002	1.0003 (0.9993 ~ 1.0013)	0.600

## 4 Discussion

According to the analysis of the and Chinese and NHANES, we found that berry consumption is associated with a lower risk of frailty. Even after adjusting for other factors that might influence the results, this association remains significant. Further analysis in the NHANES suggested that this association is more pronounced in men. Additionally, this association was observed among and overweight men who drink alcohol in this age group. Mediation analysis indicated that hemoglobin A1c mediated 6.64% of the effect.

Berries are rich in anthocyanins, dietary fiber, and ascorbic acid (vitamin C), which have been shown to have anti-inflammatory and antioxidant properties (21–23). Higher intakes of dietary flavonoids, particularly anthocyanins and flavonols (such as quercetin), are associated with a lower prevalence of frailty among American adults (24, 25). The anti-aging effects of quercetin have been confirmed through proteomics and clinical trials (26, 27). Additionally, the high levels of dietary antioxidants such as vitamins A, C (ascorbic acid), and E found in berries have also been linked to a lower prevalence of frailty in the general population (28).

It is worth noting that insufficient dietary fiber intake has been identified as a risk factor for frailty (6, 29). Previous studies have shown that elderly individuals with anemia and low dietary fiber intake have a significantly higher risk of frailty, whereas those with high fiber intake show a reduced risk (30). Berries are an excellent source of dietary fiber. Therefore, consuming berries can help reduce the risk of frailty through several mechanisms: firstly, the anthocyanins, quercetin, and various antioxidants (A, C, E) they provide can exert anti-inflammatory and antioxidant effects, combating age-related damage; secondly, their rich dietary fiber content helps compensate for the common fiber deficiency in the elderly. The combined effect of these nutrients may help alleviate key pathological changes associated with frailty, such as mitochondrial dysfunction and muscle atrophy (31, 32). Recent studies focusing on the elderly population have unequivocally demonstrated that mitochondrial dysfunction is the core driving factor behind frailty and sarcopenia (33). A review suggests that polyphenols in the diet can improve mitochondrial function by activating AMPK (34).

One key advantage of berries over other fruits is their lower glycemic index (GI), suggesting they may offer better regulation of glucose metabolism (35). Studies have shown that consuming the flesh of Brazilian berries can reduce markers of metabolic disease risk in overweight adults (36). Among elderly individuals aged 60–80 years with cardiac metabolic risk, a 24-week anthocyanin supplementation (320 mg/day) also significantly reduced inflammatory markers (CRP, IL-6) and LDL cholesterol levels (37). This is because the fat tissue of overweight individuals continuously releases pro-inflammatory factors, such as TNF-α, which exacerbates oxidative stress and chronic inflammation, leading to muscle atrophy and functional decline (38, 39). The bioactive compounds in berries, such as polyphenols, may counteract these harmful pathways by improving insulin sensitivity and inhibiting inflammatory cytokines like IL-6 (40). The endogenous antioxidant capacity of older men significantly declines with age, making them more susceptible to the vicious cycle of ‘inflammation-oxidative stress.’ Berries, rich in polyphenols like anthocyanins, can effectively break this cycle. For example, they reduce systemic inflammation by inhibiting the NF-κB pathway and boost the levels of endogenous antioxidants like glutathione (41–43).

Our study quantified the bridging role of HbA1c in the association between berry intake and frailty for the first time through a mediation model. The results showed that HbA1c mediated 6.64% of the effect, the effect proportion is small. This finding is supported by biological evidence: anthocyanins in berries can activate the AMPK signaling pathway to enhance insulin sensitivity, while high HbA1c directly drives frailty by promoting muscle protein breakdown and mitochondrial damage (44). Anthocyanins and polyphenols, abundant in berries, have been shown by multiple randomized controlled trials to enhance insulin sensitivity and reduce postprandial blood glucose fluctuations (40, 45, 46). Notably, the 6.64% mediation ratio indicates that a larger proportion of the effect is explained by other pathways. This suggests that the unique polyphenolic components in berries may play a more significant role through anti-inflammatory and antioxidant pathways. Future RCTs in pre-diabetes populations, combined with dynamic HbA1c monitoring and multi-omics analysis, are needed to further elucidate dose effects and the interaction mechanisms with non-glycemic pathways.

A study on the relationship between alcohol use and frailty among older Americans found that heavy drinking can reduce the risk of frailty in both elderly men and women (47). Individuals with regular, moderate drinking habits (as opposed to binge drinking) tend to pay more attention to the quality of their diet. Therefore, the association observed between berry consumption and physical weakness in this population may also be partially attributed to their heightened overall health awareness and healthier dietary patterns. They are more likely to adopt Mediterranean or other healthy eating patterns rich in fruits, including berries, which can provide a broader range of health benefits. Moderate drinking, particularly of red wine, which is rich in polyphenols like resveratrol, has been suggested by some studies to be beneficial for cardiovascular metabolic health (48). However, this study was designed as a cross-sectional study and could not distinguish the independent effects of berry consumption, alcohol intake behavior, and healthy lifestyle; therefore, the observed association cannot be simply attributed to alcohol consumption alone.

Although this study integrates evidence from epidemiological observations, mediation analysis, and mechanism studies, systematically elucidating the association between berry consumption and reduced frailty risk and its potential pathways, it still has several notable limitations. All these findings are based on observational designs, which make it challenging to establish a causal relationship. The temporal sequence between berry consumption and frailty cannot be established, and the possibility of reverse causality cannot be ruled out. This limitation requires validation in future studies through longitudinal cohort studies or randomized controlled trials. Binarary measurements in the China cohort may introduce non-differential misclassification bias; future studies should employ standardized dietary assessment tools to enhance cross-study comparability. There is a lack of large-scale, long-term, randomized controlled trials (RCTs) targeting different types of berries and specifically in elderly populations with frailty to directly verify their preventive or improvement effects on frailty. Future studies could simultaneously adjust for overall dietary quality scores (e.g., Health Eating Index [HEI]), specific bioactive compounds (such as flavonoids), physical activity levels, and the severity of comorbidity burden to more accurately assess the independent effects of berry consumption. The study particularly highlights the significance of the association in men. While this suggests the value of targeted interventions, it also limits the generalization of the results to broader populations, such as women, other racial/ethnic groups, and older adults.

## 5 Conclusion

In conclusion, this study demonstrates that berry consumption is independently associated with a lower risk of frailty, and this association remains statistically significant even after adjusting for other potential confounding factors. This relationship is particularly pronounced among American men. Mediation analysis revealed that glycated hemoglobin accounts for 6.64% of this association, suggesting that the negative correlation between berry intake and frailty may be partially explained by improved glycemic stability. Based on these findings, optimizing dietary quality—particularly increasing berry consumption—may contribute to reducing frailty risk in older adults, especially males; however, this conclusion requires further validation in future longitudinal studies or randomized controlled trials. Future research should also investigate alternative pathways beyond glycated hemoglobin.

## Data Availability

The original contributions presented in the study are included in the article/Supplementary material, further inquiries can be directed to the corresponding authors.
